# Sestrin2 as a Potential Target for Regulating Metabolic-Related Diseases

**DOI:** 10.3389/fendo.2021.751020

**Published:** 2021-11-03

**Authors:** Linan Gong, Zanzan Wang, Zhenggui Wang, Zhiguo Zhang

**Affiliations:** Center for Cardiovascular Medicine, First Hospital of Jilin University, Changchun, China

**Keywords:** Sestrin2, AMPK, mTOR, metabolism, metabolic diseases

## Abstract

Sestrin2 is a highly conserved protein that can be induced under a variety of stress conditions, including DNA damage, oxidative stress, endoplasmic reticulum (ER) stress, and metabolic stress. Numerous studies have shown that the AMP-activated protein kinase (AMPK)/mammalian target of rapamycin (mTOR) signaling pathway has a crucial role in the regulation of metabolism. Sestrin2 regulates metabolism *via* a number of pathways, including activation of AMPK, inhibition of the mTOR complex 1 (mTORC1), activation of mTOR complex 2 (mTORC2), inhibition of ER stress, and promotion of autophagy. Therefore, modulation of Sestrin2 activity may provide a potential therapeutic target for the prevention of metabolic diseases such as insulin resistance, diabetes, obesity, non-alcoholic fatty liver disease, and myocardial ischemia/reperfusion injury. In this review, we examined the regulatory relationship between Sestrin2 and the AMPK/mTOR signaling pathway and the effects of Sestrin2 on energy metabolism.

## Introduction

Sestrins, a family of evolutionarily highly conserved stress-induced proteins, are upregulated under oxidative stress, genotoxic stress, hypoxia, and other stress conditions (1). As stress-induced metabolic modulators, Sestrins help cells adapt to diverse stress stimuli by activating catabolic reactions, stopping anabolic activities, and initiating cell repair mechanisms, to maintain cell homeostasis (2). In mammals, there are three members of the sestrin family, Sestrins1–3, which are encoded by three independent genes, while only one Sestrin ortholog has been identified in invertebrates (3–5). Sestrin1, also referred to as p53-activated gene 26 (*PA26*), was first identified by Velasco-Miguel et al. and is a growth arrest and DNA damage-inducing gene (5). In 2002, Sestrin2, also known as hypoxia-inducible gene 95 (Hi95), was reported by Budanov et al., is highly homologous to Sestrin1, and can be induced by prolonged hypoxia and DNA damage (6, 7). Sestrin3 is directly activated by forkhead box O (FOXO) transcriptional factors (8). These three Sestrin proteins have some shared mechanisms of action, including, but not limited to, inhibiting the production of reactive oxygen species (ROS), activating AMPK, and inhibiting mTORC1 (4, 9). However, there is growing evidence that the three Sestrins behave differently and promote different biological effects *via* AMPK/mTOR signaling because they are distributed differently in different organs (10, 11). To our knowledge, Sestrin1 has an antioxidant function that can activate the AMPK signal pathway while inhibiting the mechanistic target of the mTORC1 signal pathway (12). Furthermore, Sestrin1 can be activated in a p53-dependent manner under oxidative stress in skeletal muscle, kidney, brain, and lung (7). Recent studies suggest that Sestrin1 inhibits oxidized low-density lipoprotein-induced activation of NOD-like receptor protein 3 (NLRP3) inflammasome in macrophages in a murine atherosclerosis model (12). What is even more interesting is that in multiple mouse models, Sestrin1 influences plasma cholesterol and regulates cholesterol biosynthesis (13). Among these members, Sestrin2 is the most intensively studied since its discovery in 2002. As a p53 target gene, Sestrin2 (*SESN2*) can exert cytoprotective functions in the lung, heart, liver, adipose, and kidney through activation of AMPK and inhibition of mTORC1 (6, 11, 14, 15). Furthermore, Sestrin2 is able to suppress nitric oxide release and the production of classical pro-inflammatory cytokines in cardiomyocytes (16). Sestrin3 can inhibit mTORC1 and maintain the activity of protein kinase B (AKT) *via* activating the AMPK/TSC1/2 signaling pathway (8). Sestrin3 is largely expressed in skeletal muscle, intestine, adipose, colon, and brain (17).

Increasing evidence suggests that that Sestrin2 has two main biological functions. Through its own oxidoreductase activity or activation of antioxidant damage related pathways, Sestrin2 can reduce the damage of oxidative stress to protect cells and tissues and maintain redox homeostasis (18, 19). In addition to its redox activity, Sestrin2 can also inhibit the mammalian target of mTORC1 through AMPK-dependent or -independent pathways (20). These two activities of human Sestrin2 (hSestrin2) are supported through its two separate domains, which were determined from X-ray crystallographic studies. A recent study of the X-ray crystal structure of hSestrin2 showed that it consists of well-conserved Sesn-A, Sesn-B, and Sesn-C domains (11). Sesn-A and Sesn-C are structurally similar but functionally distinct from each other (21). Sestrin2 controls ROS and mTOR signaling through two separate functional domains (22). While Sesn-A reduces alkyl hydroperoxide radicals through its helix–turn–helix oxidoreductase motif, Sesn-C modifies this motif to accommodate physical interactions with GAP activity towards Rags 2 (GATOR2) and subsequent inhibition of mTORC1 (21, 23). Sestrin2 has a significant role in the inhibition of ER stress and the activation of autophagy and is considered to improve obesity-induced and age-related pathologies by inhibiting mTORC1 (15, 24). Therefore, Sestrin2 may represent a novel class of potential targets for the therapeutic intervention of metabolic diseases. In this review, we discuss the regulatory relationship between Sestrin2 and AMPK/mTOR signaling and the effects of Sestrin2 on energy metabolism.

## Regulation of Sestrin2 Expression in Response to Diverse Stress Conditions

Human beings exist in a constantly changing environment and face frequent challenges that threaten our survival and health. In response to stress, the body undergoes very subtle changes at the cellular and molecular level. Understanding how Sestrin2 is regulated under different stress conditions is very helpful for us in studying Sestrin2. Therefore, it is of great significance to study the regulatory mechanism of Sestrin2 expression under different types of stress conditions.

### Sestrin2 and Oxidative Stress

**Figure 1:**
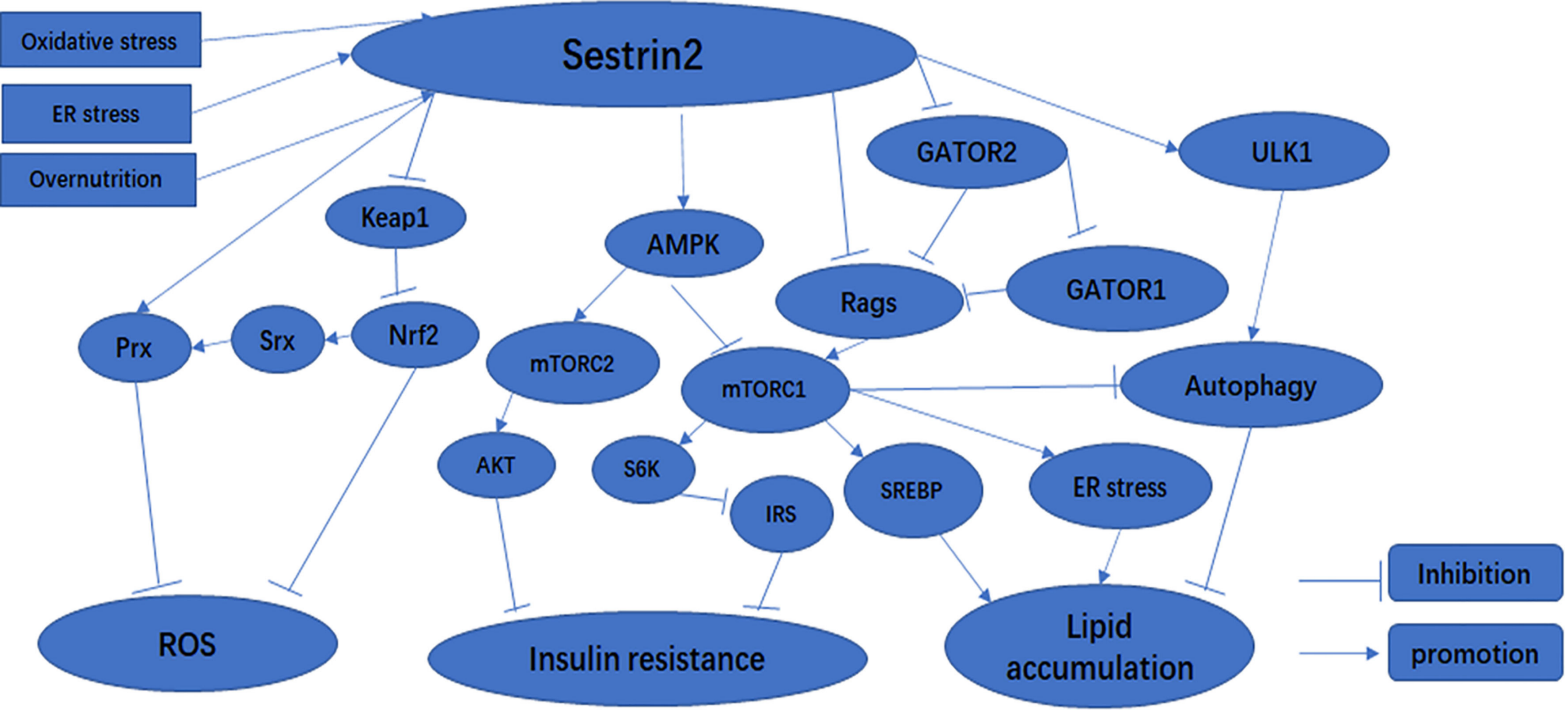
The effect of Sestrin2 on metabolic-related signaling pathways.

Reactive oxygen species and reactive nitrogen species (RNS) are generated continuously in the body through oxidative metabolism, biological functions of mitochondria, and immunologic functions (25). Physiological ROS are crucially important for intracellular and extracellular signal transduction (26). However, it is well-known that overloaded ROS and RNS can bind with and destroy most cellular biomolecules (lipids, enzymes, sugars, proteins, nucleic acids, and other small molecules) under oxidative stress (27–29). Oxidative stress is considered to be an imbalance in redox properties in certain cellular environments (30), and plays a crucial role in the development of numerous human diseases, including diabetes, obesity, and myocardial injury (31–33). Resistance to oxidative stress injury is one of the important functions of Sestrin2. In response to oxidative stress, the expression of Sesrin2 is regulated at the mRNA and protein level by various transcription factors, including nuclear factor kappa-B (NF-κB), activator protein-1 (AP-1), CCAAT-enhancer-binding protein beta (C/EBPβ), forkhead box O3 (FOXO3), and p53 (19, 24, 34–36). Sestrin2 has been suggested to maintain the balance of oxidative metabolism through two main biological functions. First, as an antioxidant enzyme, Sestrin2 is capable of directly reducing the accumulation of ROS (37). However, the intrinsic catalytic antioxidant activity of Sestrin2 remains elusive and limited. Second, recent studies have demonstrated that Sestrin2 inhibits ROS production and defends cells against oxidative stress, which is likely to be mainly attributed to its regulation of several signaling pathways related to oxidative stress: the Kelch-like Ech-associated protein 1 (KEAP1)/NF-E2 related factor-2 (NRF2) antioxidant signaling pathway (2) (**Figure 1**) and the AMPK and mTORC1 pathways (which will be described in detail later) (38). NRF2 is a transcription factor that can bind to antioxidant-responsive elements (AREs) to promote the expression of many antioxidant molecules to protect cells from oxidative insults (36). NRF2 is constitutively expressed in the cytoplasm under physiological conditions (39). Under normal conditions, KEAP1 binds to NRF2, preventing NRF2 translocation to the nucleus, promoting its ubiquitination and proteasome degradation, and maintaining free NRF2 in the cytoplasm at a low level (19). Under oxidative stress, NRF2 dissociates from KEAP1 and translocates to the nucleus. NRF2 binding to ARE activates the transcription of target genes *PRX*, *SRX*, superoxide dismutase (*SOD*), catalase (*CAT*), heme oxygenase 1 (*HO1*), and glutathione peroxidase 1 (*GPX1*) (40, 41). In cellular studies, it was found that Sestrin2 binds to unC-51-like kinase 1 (ULK1) and p62 to form functional complexes, and that Sestrin2 promotes the phosphorylation of p62, which further promotes KEAP1 degradation and NRF2 activation (42). In addition, in studies of liver damage caused by oxidative stress, Sestrin2 was shown to act as a scaffold protein to enhance the weak binding of KEAP1 to p62, thereby promoting KEAP1 autophagy degradation and preventing oxidative liver injury (43). More interestingly, NRF2 regulates the expression of Sestrin2 by binding to the ARE promoter of *SESN2* under oxidative stress (37). A positive feedback loop is formed between Sestrin2 and NRF2 to promote the transcription and translation of antioxidant-related genes downstream of NRF2 and to protect cells from oxidative damage (36). Therefore, during oxidative stress, Sestrin2 is crucial to maintaining cellular homeostasis.

### Sestrin2 and Endoplasmic Reticulum Stress

ER stress occurs when unfolded or misfolded proteins accumulate in the endoplasmic network lumen due to adverse physiological conditions (44). During ER stress, cells can improve their protein folding ability, inhibit protein production and accumulation, induce ER stress-related gene transcription, and strengthen the self-repair ability of ER to restore protein-folding homeostasis and regulate ER homeostasis through a series of transduction pathways, including protein kinase R-like endoplasmic reticulum kinase-eukaryotic translation-initiation factor 2α (PERK-eIF2α), inositol-requiring enzyme 1a-X-box-binding protein 1 (IRE1-XBP1), and activating transcription factor 6-CREBH (ATF6-CREBH). These reactions are called the unfolded protein reaction (UPR) (45). If ER stress is too strong or lasts too long, these responses are not enough to restore ER homeostasis, and apoptosis is eventually induced (46). A growing body of research has demonstrated that expression of Sestrin2 can be upregulated under ER stress conditions (15, 35, 47). For instance, Park et al. (15) found that upregulated Sestrin2 is associated with an ER stress-activated transcription factor, CCAAT enhancer-binding protein beta (c/EBPb). Once induced, Sestrin2 in turn stops protein synthesis by inhibiting mTORC1. Recently, a study by H. Jeong Kim et al. (48) revealed that induction of Sestrin2-regulated genes can be connected *via* activation of the PERK/eIF2α/ATF4 pathway. Consistent with these findings, Jegal et al. (35) demonstrated that under ER stress, expression of Sestrin2 can be enhanced *via* activating transcription factor 6 in hepatocytes, and Sestrin2 decreases the phosphorylation of JNK and p38 as well as PARP cleavage, and blocks the cytotoxic effect of excessive ER stress so as to play a hepatoprotective role both *in vitro* and *in vivo*. Furthermore, Ding et al. (49) elucidated that upregulation of Sestrin2 expression is dependent on ATF4 and NRF2 but not p53 under ER stress induced by glucose starvation. To summarize, Sestrin2 might serve as an important regulator that exerts cell and tissue protection functions under excessive ER stress. However, the exact mechanism by which ER stress induces Sestrin2 expression remains poorly understood and requires further exploration.

### Sestrin2 and Obesity

Obesity is traditionally considered to be the excessive accumulation of fat in the body, which is a serious hazard to human health, and in clinical practice, obesity is usually assessed by the body mass index (BMI) (50). With the improvements in the general standard of living, the incidence of obesity has risen sharply (51). Obesity is a major contributor to the development of metabolic syndromes, including type 2 diabetes mellitus, hypertension, hyperlipidemia, and cardiovascular disease (52). Studies have shown that overnutrition and a sedentary lifestyle are the main causes of obesity (53). mTORC1 is a nutrient-sensitive protein kinase that has a fundamental role in maintaining metabolic homeostasis (54). Recent research has clarified that overnutrition can result in chronic mTORC1 activation (55). In response to persistent overnutrition, chronic mTORC1 activation can enhance protein and lipid biosynthesis and inhibit autophagic catabolism (56). Several studies confirmed that chronic mTORC1 activation mediated by stress responses such as overnutrition ultimately leads to overexpression of Sestrin2 (24, 57). Lee et al. (24) found that the expression of *Drosophila* Sestrin (dSesn) is upregulated upon chronic mTORC1 activation *via* the c-Jun N-terminal kinase (JNK) and FOXO signaling pathways. Loss of dSesn results in triglyceride accumulation and mitochondrial dysfunction. Furthermore, another study by Lee et al. (57) demonstrated that Sestrin2 is the only Sestrin protein that is induced by overnutrition and obesity and attenuates chronic mTORC1 activation *via* the mTORC1/S6K axis in mouse liver. In agreement with these conclusions, Kimball et al. (58) revealed that Sestrin2 expression was upregulated in the livers of rats fed with a high-fat diet. Thus, in a nutshell, Sestrin2 exerts important metabolic homeostatic functions.

## Sestrin2 and the AMPK/mTOR Signaling Pathway

mTOR is an evolutionally conserved protein that is a critical regulator of cell proliferation, proliferation, metabolism, and autophagy (37). mTOR promotes anabolic processes such as ribosome biogenesis and synthesis of proteins, nucleotides, fatty acids, and lipids, and inhibits catabolic processes such as autophagy (54). It is composed of two structurally and functionally distinct complexes, mTORC1 and mTORC2, which are characterized by the presence of Raptor and Rictor, respectively (59). mTORC1 consists of mTOR Raptor, PRAS40,and mLST8, while mTORC2 is composed of mTOR, Rictor, Sin1, Protor, and mLST8 (54). mTORC1 promotes protein and lipid synthesis through the phosphorylation of its distinctive substrates, such as ribosomal protein S6 kinase (S6K) and eukaryotic initiation factor 4E-binding protein 1 (4EBP1) (2). In addition, mTORC1 may also regulate adipogenesis through the regulation of the sterol regulatory element-binding proteins (60). Furthermore, mTORC1 can phosphorylate and suppress autophagy-initiating protein kinases unc-51-like kinase 1 (ULK1) to inhibit cellular autophagic catabolism (61). mTORC2 regulates metabolism and cytoskeletal tissue in response to growth factors through the activation of AGC family kinases, including AKT, SGK1, and PKCα (62). Recent studies have shown that mTORC2 in particular is a crucial controller of lipid metabolism that regulates adipogenesis in the liver (60).

AMPK, an important nutrient-sensing protein kinase, has a critical role in increased catabolism and decreased anabolism (63). AMPK can inhibit the phosphorylation of the acetyl-CoA carboxylases ACC1 and ACC2, HMG-CoA reductase, and the glycogen synthases GYS1 and GYS2 to regulate the biosynthesis of glycogen and lipids (63). It can also inhibit mTORC1 activity through the phosphorylation of its regulatory subunit Raptor (64) or through the phosphorylation of tuberous sclerosis complex 2 and inhibition of mTORC1-activating guanosine triphosphatase (GTPase) Rheb (4). In addition, AMPK restrains the transcriptional activity of sterol regulatory element binding protein (SREBP) through direct phosphorylation to decrease the expression of lipogenic genes (65).

Once induced by stress, Sestrin2 affects a variety of signaling pathways, thus upregulating stress adaptation mechanisms (23). When induced in response to oxidative stress, Sestrin2 inhibits mTORC1 through the activation of AMPK (66). Consequently, Sestrin2-deficient cells and tissues exhibit lower AMPK and higher mTORC1 activity under both normal and stressed conditions (6, 24, 66). It has been reported that Sestrin2 acts as a scaffold protein, promoting the binding of LKB1 to AMPK and subsequent AMPK phosphorylation and activation, and controls mTORC1 signaling as an inhibitor of guanine nucleotide dissociation in Rag GTPases (6, 67, 68). Sestrin2 can also activate AMPK through direct interaction with the α subunit of the AMPK complex (66).Recent studies have shown that Sestrin2 can inhibit mTORC1 through AMPK-dependent or -independent pathways (15, 20, 57, 68) (**Figure 1**). Sestrin2 can also modulate amino acid-stimulated mTORC1 activation through direct interactions with Rag A/B GTPases or GATOR2 complexes (68, 80). Sestrin2 binds to GATOR2 and releases GATOR1 from GATOR2-mediated inhibition. Released GATOR1 subsequently binds to and inactivates RagB, ultimately resulting in mTORC1 suppression (81) (**Figure 1**). In addition, Sestrin2 plays a critical role in the activation of autophagy through multiple mechanisms including activation of AMPK, inhibition of mTORC1, and activation of ULK1 (82) (**Figure 1**). Therefore, the AMPK/mTORC1 signaling pathway is critical for Sestrin2 in controlling cell metabolism and survival under stress conditions (**Figure 1**).

## Role of Sestrin2 in Diabetes, Non-Alcoholic Fatty Liver Disease, and Myocardial Ischemia/Reperfusion (I/R) Injury

Mounting evidence has demonstrated that Sestrin2 is upregulated in response to diverse stress conditions, including oxidative stress, ER stress, and metabolic stress. Sestrin2 exerts a significant influence on the protection of human cells and tissues *via* related signal transduction pathways, and was shown to play a critical role against metabolic diseases, such as diabetes, obesity-related non-alcoholic fatty live, and myocardial I/R injury (**Table 1**).

**Table 1:** Summary of the role of Sestrin2 in metabolic diseases.

Disease	Signaling pathway	Effect	Reference
Diabetes	AMPK/mTOR	Improves insulin resistance	(57, 69)
		Increases insulin-sensitivity	
Nonalcoholic fatty liver disease	AMPK/mTORC1	Reduces lipid synthesis	(24, 70)
		Attenuates ER stress	(15)
	Nrf2/ Keap1	Promotes autophagy	(2)
	Nrf2/HO-1	Prevents oxidative liver damage	(43)
	JNKs	Keeps redox balance	(71)
		Attenuates lipotoxicity	(72)
Myocardial ischemia/reperfusion (I/R) injury	AMPK/PGC-1α	Reduces the area of myocardial injury Attenuates the sensitivity of myocardium to ischemia	(73)
	AMPK/LKB1	Protects mitochondrial biogenesis	(74)
		Inhibits myocardial cell apoptosis	(75)
	MAPK signaling pathway	Diminishes myocardial infarct size	(76)
	Antioxidant protein	Improves function of infarcted myocardium	(77)
	AMPK/mTOR	Refines myocardial substrate metabolism	(78)
		Modulates cardiac inflammation Restrains ROS production Improves contractile function Attenuates myocardial hypertrophy	(79)
		Improves cardiac function	

### Sestrin2 and Diabetes

Diabetes is the most common metabolic disease, and is a chronic disease characterized by persistent hyperglycemia (83). More than 90% of diabetics have type 2 diabetes, and insulin resistance is consistently found in patients with type 2 diabetes (84). Insulin resistance is an impaired biological response to insulin stimulation in target tissues, primarily liver, muscle, and adipose tissue (85). Insulin resistance impairs glucose processing, leading to a compensatory increase in beta cell insulin production and hyperinsulinemia. Sestrin2 is highly expressed in the liver (86). According to literature reports, two pathways of Sestrin2 affect cell signaling pathway transduction: one activates the AMPK pathway and the other downregulates the mTOR pathway (2, 87). AMPK is an enzyme activated in energy-deficient conditions (2). Sestrin2 is induced by oxidative stress through activation of the NRF2 and JNK/AP-1 signaling axes (4, 43, 88). In bacteria, AhpD is a critical member of the antioxidant defense system and regenerates peroxide AhpC, a bacterial peroxidant protein (Prx), through catalytic reduction. In mammalian cells, Sestrins interact with overoxidized PRX and promote its regeneration. Here, Sestrins act similarly to AhpD in bacteria (77). Sestrins have no direct catalytic activity leading to the reduction of PRX, but may regenerate PRX by promoting the activity of other oxidoreductases, such as thioredoxin (SRX) (4). Sestrins can increase SRX expression by activating NRF2 (6, 43) (**Figure 1**). Increased glucose downregulates Sestrin2 expression, thereby increasing mTOR activity and inhibiting AMPK (87, 89). Moreover, when treated with high levels of glucose, such as metformin (an AMPK agonist and mitochondrial respiratory inhibitor), Sestrin2 was upregulated, mTOR activity was significantly increased, and AMPK activity was decreased (6, 87). S6K is an effector of the mTOR pathway (90). By activating S6K, mTORC1 promotes insulin resistance by inhibiting phosphorylation of insulin receptor substrates (IRS) (**Figure 1**) and attenuating the insulin-induced phosphatidylinositol 3-kinase (PI3K)/AKT signaling pathway (6). mTORC1/S6K activity leads to serine phosphorylation and protein degradation of IRS, forming a negative feedback loop in which insulin signaling attenuates subsequent insulin action (89). Lack of amino acids, especially leucine, leads to rapid dephosphorylation of the mTORC1 effectors S6K and 4EBP1, which depend on mTORC1 for amino acid resynthesis (91). Sestrin2 is required to maintain insulin sensitivity in the liver in high-fat diet (HFD)-induced dietary obesity and Lepob mutation-induced inherited obesity (57). AMPK and mTORC1 are important protein kinases with complete antagonistic functions in metabolic homeostasis (92). Sestrin1 and Sestrin2 activate AMPK through direct interaction with the α subunit of the AMPK complex (66). Sestrin2 acts by activating AMPK and inhibiting various mechanisms of mTORC1. We know that AMPK and mTORC1 play critical roles in metabolism, and Sestrin2 is involved in many biological processes as an upstream regulator of AMPK and mTORC1 kinases (57) (**Figure 1**). Previous experiments in liver-specific *Sesn3* transgenic mice and knockout mice showed that the transgenic mice were protected against insulin resistance induced by a high-fat diet, while the *Sesn3* knockout mice showed metabolic defects such as insulin resistance and glucose intolerance (93, 94). Therefore, we can recognize that Sestrin2 is a potential insulin sensitizer, and that Sestrin deficiency and/or dysfunction may lead to insulin resistance, which can lead to the development of diabetes. Sestrin2 may be a potential therapeutic target for metabolic diseases such as diabetes (82).

### Sestrin2 and Non-Alcoholic Fatty Liver Disease

With the global trend in obesity and its related metabolic syndromes, non-alcoholic fatty liver disease (NAFLD) has become an important cause of chronic liver disease in developed countries (95). NAFLD is the hepatic manifestation of metabolic syndrome characterized by intracellular excessive accumulation of lipids in hepatocytes, excluding alcohol and other damaging factors (95). NAFLD involves a range of liver pathological changes, including steatosis, steatohepatitis, advanced fibrosis, and cirrhosis (96). Existing studies have shown that NAFLD is closely associated with persistent ER stress, inhibition of autophagy, mitochondrial dysfunction, insulin resistance, lipotoxicity, and overnutrition (15, 71, 96, 97). Overnutrition and obesity give rise to excessive lipid accumulation in hepatocytes, known as hepatic steatosis (98). We have previously shown that overnutrition can lead to chronic mTORC1 activation (53). mTORC1 can intensify the transcriptional activity of sterol regulatory element binding protein (SREBP) and the expression of lipogenic genes to enhance lipid synthesis (**Figure 1**). It is evident that chronic mTORC1 activation along with persistent inhibition of autophagy attenuates clearance of liver lipid droplets, ultimately leading to hepatosteatosis (99). As a feedback inhibitor of mTORC1, Sestrin2 can partially ease the effect of chronic mTORC1 activation. For instance, loss of dSesn leads to moderate downregulation of AMPK and upregulation of dTORC1 in *Drosophila*, which contributes to the increased expression of liposomal enzyme genes and ultimately to the accumulation of triglycerides (24). Similarly, a study has confirmed that hepatosteatosis is more serious, and that the primary cause of hepatosteatosis is reduced lipid β-oxidation due to reduced autophagy or mitochondrial biogenesis, rather than increased adipogenesis in Sestrin2-deficient liver (57). Furthermore, Sestrin2 also reduces the susceptibility of the liver to oxidative damage *via* the NRF2/KEAP1 signaling pathway (43). In mice with Sestrin2 deficiency, cells continue to translate large amounts of proteins during ER stress, which subsequently leads to extensive liver damage, inflammation, and fibrosis (15). Accordingly, once induced by ER stress, Sestrin2 maintains endoplasmic reticulum homeostasis by inhibiting the AMPK/mTORC1 signaling pathway (**Figure 1**), thereby protecting against hepatosteatosis (15). Kim et al. found that carbon monoxide can induce Sestrin2 upregulation, and Sestrin2 protects against hepatosteatosis by activating autophagy through the AMPK/mTORC1 axis in a cellular model of NAFLD (48). More interestingly, Sestrin2 plays an important role in the protection against lipotoxicity-associated oxidative stress in the liver *via* suppression of JNKs (72). In summary, Sestrin2 has a significant impact on lipid metabolism and represents a potential therapeutic strategy for NAFLD.

### Sestrin2 and Myocardial Ischemia/Reperfusion Injury

Coronary heart disease, also known as ischemic heart disease (IHD), refers to the interruption of blood flow to the heart muscle due to atherosclerosis, coronary thrombosis, and narrowing of the small arteries of the heart, which remains the leading cause of death worldwide because the incidence of IHD increases with age (100, 101). After an acute myocardial infarction, although early and successful myocardial reperfusion through thrombolytic or percutaneous coronary intervention is the most effective way to rescue the ischemic heart and improve the clinical outcome, the recovery of blood flow can result in myocardial injury, which reduces the efficacy of myocardial reperfusion, namely ischemia/reperfusion (I/R) injury (78, 102). Myocardial I/R injury is closely related to ROS, calcium overload, energy metabolism disorders, acidosis, and inflammation (102). Some studies have reported that the I/R process usually results in elevated levels of ROS production, especially in the early stages of reperfusion, directly causing myocardial injury (103). Moreover, excessive ROS leads to programmed cell death through the activation of the mitogen-activated protein kinase signaling pathway (104). Mitochondria have an important role in ROS degradation, and dysfunctional mitochondria are the main sources of pathological ROS (105, 106). AMPK can protect mitochondria and play an antioxidant role during the I/R process (78). Furthermore, AMPK has an essential role in the activation of glucose uptake in the ischemic heart (107–109). AMPK also activates 6-phosphofructo 2-kinase, which leads to the production of fructose 2, 6-bisphosphate, further promoting glucose utilization in the ischemic heart (75, 110, 111). Therefore, AMPK is a protein kinase with significant cardiac protection against myocardial I/R injury (74). Sestrin2 has been shown to increase the activation of AMPK *via* interactions with LKB1 to improve myocardial substrate metabolism under I/R stress (75). Sestrin2 was originally characterized as a critical antioxidant protein that contributes to cycling of peroxiredoxins (77). Independent of this redox-regulating activity, Sestrin2 can modulate the activation of AMPK to maintain the integrity of mitochondrial function and reduce the generation of ROS (14, 66, 74, 112). A study by Quan et al. (76) revealed that Sestrin2 greatly reduces myocardial damage by modulating inflammation and redox homeostasis in mouse hearts during I/R stress. Hence, Sestrin2 provides cardioprotection by repressing ROS during I/R injury. Furthermore, Quan et al. (73) found that the decreased Sestrin2 levels in aging and *Sesn2*-knockout mice led to increased sensitivity to ischemic insults and areas of myocardial injury, which aggravated worsened cardiac dysfunction. Sestrin2 protects mitochondrial function by activating the AMPK/PGC-1α signaling pathway during myocardial ischemia (73). Sestrin2 has been shown to be upregulated under anoxic and ischemic conditions and has a protective role against myocardial ischemia (7, 74, 78). The loss of Sestrin2 aggravates the accumulation of fatty acids, thereby altering substrate metabolism in the heart and increasing the production of ROS (37, 78). Inactivation of the *SESN* gene in invertebrates can lead to a variety of metabolic diseases such as muscle degeneration, oxidative damage, fat accumulation, and mitochondrial dysfunction (4). Existing studies have reported that Sestrin2 is involved in the protection of cardiovascular disease by regulating the AMPK signaling pathway (38). Sestrin2 protein accumulates in the heart during myocardial ischemia (17), and the myocardial infarction area in *Sesn2* knockout mice was significantly larger than that in wild-type mice when myocardial ischemia reperfusion occurred (74). In conclusion, Sestrin2 has an influential role in cardioprotection during myocardial I/R injury. Therefore, Sestrin2 may be a therapeutic target for cardiovascular disease, potentially revealing a new avenue of investigation for the treatment of cardiovascular diseases.

## Problems and Prospects

Sestrin2 is a critical intracellular sensor that activates AMPK and inhibits mTORC1 to regulate autophagy, ER stress, inflammation, metabolic homeostasis, and oxidative stress. Clearly, the AMPK/mTORC1 axis is regulated by Sestrin2 and it provides the main channel for its function. Sestrin2 regulates metabolism-related signaling pathways, as summarized in **Figure 1**. However, despite their physiological relevance, the exact mechanism by which Sestrin2 promotes AMPK activation remains unclear. Therefore, further studies are needed to determine the detailed molecular function of Sestrin2.

Evidence suggests that Sestrin2 has an important clinical function in responding to a variety of metabolic diseases, such as diabetes mellitus, insulin resistance, and lipid metabolism disorders. In recent studies, serum Sestrin2 levels were significantly reduced in obese children and patients with diabetic nephropathy (113, 114). This suggests that the expression or secretion of Sestrin2 is somewhat obstructed in the disease state. Furthermore, a study by Kim et al. revealed that in NAFLD cell models, carbon monoxide protects the liver against steatosis by inducing upregulation of Sestrin2, which activates autophagy through the AMPK/mTORC1 axis (48). Consistent with this view, as a glucagon-like peptide 1 (GLP-1) analog, liraglutide could reverse NAFLD by enhancing the level of Sestrin2 protein and the Sestrin2-mediated NRF2/HO-1 pathway (71). Therefore, we hypothesized that upregulation of Sestrin2 expression could ameliorate metabolism-related diseases. Sestrin2 shows great potential as a good prognostic marker and a viable therapeutic target in a variety of diseases. However, how to induce Sestrin2 upregulation remains elusive under different disease conditions. To design therapeutic strategies to upregulate Sestrin2, it is important to further study the upstream and downstream pathways of the multipotent beneficial effects of Sestrin2. Future studies should use transgenic animal models with conditional organ-specific knockout of *Sesn2* and attempt to link Sestrin2 levels to disease progression, which will help us identify biochemical pathways regulated by Sestrin2 in specific diseases.

## Author Contributions

LG and ZZW worked out the theme and content of the article. ZGW completed the production of charts. ZZ reviewed and revised the full text. All authors contributed to the article and approved the submitted version.

## Conflict of Interest

The authors declare that the research was conducted in the absence of any commercial or financial relationships that could be construed as a potential conflict of interest.

## Publisher’s Note

All claims expressed in this article are solely those of the authors and do not necessarily represent those of their affiliated organizations, or those of the publisher, the editors and the reviewers. Any product that may be evaluated in this article, or claim that may be made by its manufacturer, is not guaranteed or endorsed by the publisher.
